# Relationships between Objectively Measured Sedentary Behavior during Pregnancy and Infant Birthweight

**DOI:** 10.3390/ijerph181910000

**Published:** 2021-09-23

**Authors:** Abdelmoumene Benabid, Lara Deslauriers, Isabelle Sinclair, Myriane St-Pierre, Cathy Vaillancourt, Sonia Gagnon, Kelsey N. Dancause

**Affiliations:** 1 Département des Sciences de L’activité Physique, Université du Québec à Montréal (UQAM), Montreal, QC H2X 1Y4, Canada; benabid.abdelmoumene@courrier.uqam.ca (A.B.); lara.deslauriers.16@gmail.com (L.D.); sinclair.isabelle@courrier.uqam.ca (I.S.); myrianest@hotmail.com (M.S.-P.); 2 INRS-Centre Armand Frappier Santé Biotechnologie, Laval, QC H7V 1B7, Canada; Cathy.Vaillancourt@inrs.ca; 3 Réseau Intersectoriel de Recherche en Santé, Université du Québec (RISUQ), Québec, QC G1K 9H7, Canada; 4 Département D’obstétrique-Gynécologie, Hôpital du Sacré-Coeur de Montréal, Université de Montréal, Montreal, QC H4J 1C5, Canada; sonia.gagnon.2@umontreal.ca

**Keywords:** physical activity, sedentarity, health behaviors, developmental origins of health and disease, maternal and infant health

## Abstract

Background: Although numerous studies have assessed physical activity during pregnancy and relationships with infant outcomes, such as birthweight, few have evaluated sedentary behavior. Our objective was to evaluate sedentary behavior across pregnancy and relationships with infant birthweight in a sociodemographically diverse sample. Methods: We measured device-assessed sedentary behavior and physical activity over three days at 16–18, 24–26, and 32–34 weeks gestation and infant birthweight from medical records among 71 participants. We used linear regression to assess relationships between sedentary behavior at each evaluation period with birthweight-for-gestational age Z-scores (BW-for-GA). Results: There were no linear relationships between sedentary behavior and BW-for-GA at any evaluation period. We observed a modest curvilinear relationship between sedentary behavior at 16–18 weeks and BW-for-GA (R^2^ = 0.073, *p* = 0.021). Low and high levels of sedentary behavior predicted lower BW-for-GA. Multivariate models suggested that this relationship was independent of physical activity levels. Conclusions: Considering the high levels of sedentary behavior during pregnancy observed in many studies, even modest associations with birthweight merit further consideration. Relationships might not be evident later in pregnancy or if only linear relationships are considered. More detailed studies could help guide recommendations on sedentary behavior during pregnancy and the development of more comprehensive interventions.

## 1. Introduction

Physical activity (defined as “any bodily movement produced by skeletal muscles that results in energy expenditure” [1]) during pregnancy contributes to numerous maternal health benefits, such as reduced risk of excessive gestational weight gain, gestational diabetes, and symptoms of postpartum depression [2]. Physical activity during pregnancy also holds benefits for birth and infant outcomes [3], such as reduced risk of preterm birth [4] and macrosomia [5]. As such, the U.S. Department of Health and Human Services [6], the Canadian Society for Exercise Physiology [7,8], and other international organizations, such as the World Health Organization [9], encourage pregnant women to participate in at least 150 min of moderate-intensity physical activity each week, and the American College of Obstetricians and Gynecologists (ACOG) recommends that women with uncomplicated pregnancies engage in 20 to 30 min of physical activity per day on most days or every day of the week [10].

Although the benefits of and recommendations for physical activity during pregnancy are widely discussed, those for sedentary behavior (defined as “any waking behavior characterized by an energy expenditure ≤1.5 metabolic equivalents while in a sitting, reclining, or lying posture” [11]) are less clear, and few guidelines exist [3]. Canadian guidelines highlight the importance of beginning physical activity for pregnant women who had been previously inactive, but “inactive” is not defined, and sedentary behavior is not specifically discussed [7]. Similarly, the ACOG notes that “pregnant women who were sedentary before pregnancy should follow a more gradual progression of exercise”, but sedentarity is not further discussed or defined [10]. The lack of specific advice is not a reflection of the importance of sedentary behavior but rather of the lack of research on sedentary behavior specifically. Physical activity guidelines from the United Kingdom (UK) [12] note that there are insufficient data to make concrete suggestions on sedentary behavior thresholds for adults or levels of physical activity necessary to mitigate negative effects of sedentary behavior.

Given the importance of sedentary behavior on health in the general population and the potential for sedentary behavior during pregnancy to impact not only maternal but also infant health outcomes, more studies of sedentary behavior during pregnancy are needed. Our objective was to analyze relationships between device-assessed sedentary behavior and infant birthweight among a sociodemographically diverse sample in Canada. Specifically, we aimed to evaluate patterns of sedentary behavior over the course of pregnancy and to evaluate links between sedentary behavior and infant birthweight. Furthermore, we aimed to evaluate whether potential relationships between sedentary behavior and birthweight were independent of physical activity patterns. Considering the paucity of data on sedentary behavior during pregnancy and infant birthweight, such studies are important to inform the development of more concrete recommendations on the practice of sedentary behavior during pregnancy.

## 2. Methods

This project was approved by the Research Ethics Committee of the Centre Intégré Universitaire en Santé et Services Sociaux du Nord de l’île de Montréal. All participants provided written informed consent.

### 2.1. Sample

We recruited 81 women with singleton pregnancies in their first trimester for studies of stress and health behaviors during pregnancy from February 2017–December 2017. Recruitment was through the Department of Obstetrics and Gynecology at the Hôpital du Sacré-Coeur and associated clinics, via flyers distributed by obstetricians and posted in waiting rooms. Exclusion criteria included multiple gestation, in-vitro fertilization, cardiovascular conditions, plans to move away before delivery, and inability to read and complete questionnaires in English or French. We collected data three times during pregnancy, at 16–18, 24–26, and 32–34 weeks gestation. Each assessment included three days of data collection, typically weekdays. Researchers met participants at their homes or a place of their choosing to deliver the study materials and returned to collect them after three days.

Of the 81 participants, five suffered pregnancy loss and were thus not included in the current analyses. Four had incomplete data on sedentary behavior, physical activity, or infant birthweight. Finally, we removed one participant who delivered prematurely before the third data collection. The current sample thus includes 71 women with data on objectively measured sedentary behavior and physical activity at all evaluations. No participants had medical contraindications for the practice of physical activity.

### 2.2. Variables

Key variables included sedentary behavior (sitting time, hours per day) and physical activity (steps per day). We used the Polar V800 watch (Polar Canada, Lachine, QC, Canada), which has an integrated accelerometer, to collect data. Mean sitting time and steps per day over the course of the three days was computed for each evaluation period and used in analyses. We used questionnaires to assess sociodemographic characteristics, including participants’ age, education, income, country of origin, ethnicity, and maternal and pregnancy characteristics, such as number of children and due date. Annual household income was assessed using 10 categories ranging from <$10,000 CAD to >$250,000 CAD. This was re-classified into three categories (<$20,000, $20,000 to $50,000, and >$50,000 CAD) for descriptive statistics. Education was assessed using seven categories, from “Secondary not completed” to “Post-doctorate”, and years of education were computed for each participant. This was re-classified into three categories (secondary or less, college, and university or higher) for descriptive statistics. Infant birthweight (grams) and gestational age at birth (weeks) were collected from medical records following delivery. We computed sex- and gestational age-specific birthweight Z-scores (birthweight-for-gestational age Z-scores, BW-for-GA) based on Canadian references [13]. We chose BW-for-GA as the dependent variable to facilitate comparison of birthweights for infants with different gestation lengths and contextualization with regard to the general Canadian population.

### 2.3. Analyses

We analyzed descriptive statistics, including means and standard deviations or frequencies, for each variable. Given potential seasonal differences in activity patterns, we compared mean physical activity and sedentary behavior across seasons (winter, spring, summer, and fall) at each assessment period. We used repeated measures ANOVA to test changes in sedentary behavior and physical activity over the course of pregnancy.

We used linear regression to test relationships between sedentary behavior and physical activity with BW-for-GA. We first tested linear relationships between sedentary behavior or physical activity and BW-for-GA, then entered a quadratic term for sedentary behavior or physical activity to test potential curvilinear relationships. Analyses were repeated for each of the three assessment periods.

To validate significant relationships, we evaluated multivariate models of relationships between sedentary behavior or physical activity and BW-for-GA, controlling for key covariates, including maternal age, number of children, education, and income. Given the diverse sample, we also included immigration status (immigrant or non-immigrant) and ethnicity (visible minority, referring to persons other than Aboriginal peoples who are non-Caucasian in race or non-white in color [14], or not a visible minority). We then conducted a second model including both physical activity and sedentary behavior, controlling for covariates. Analyses were conducted using SPSS version 27.0 (IBM Corp., Armonk, NY, USA).

We validated models removing one outlier with severe macrosomia and one outlier with very high levels of physical activity at the first evaluation (described below). Results were unchanged. We thus present analyses with the full sample here.

## 3. Results

Sample characteristics are shown in Table 1. Mean steps per day and hours of sedentary behavior per day were consistent with observations from other studies with healthy pregnant women. There were no significant differences among seasons for mean steps per day (Evaluation 1, *p* = 0.467; Eval. 2, *p* = 0.146; Eval. 3, *p* = 0.276) or sedentary behavior (Eval. 1, *p* = 0.812; Eval. 2, *p* = 0.408; Eval. 3, *p* = 0.584) (full data not shown).

Birthweight-for-gestational age Z-scores (BW-for-GA) ranged from −2.47 to 3.69. Most values were within normal-for-gestational age ranges. Two infants had low birthweight (birthweight < 2500 g), four were small for gestational age (birthweight < 10th percentile or Z-scores less than −1.28), and five had macrosomia (birthweight > 4000 g).

**Figure 1:**
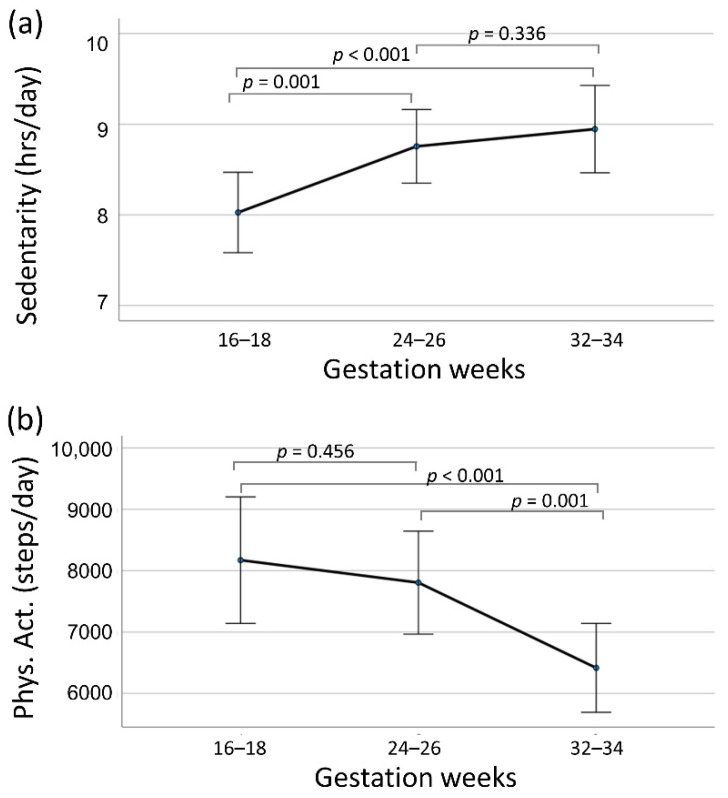
Mean sedentary behavior (**a**) and physical activity (**b**) at each evaluation period. Bars represent 95% confidence intervals.

Repeated measures ANOVA indicated that levels of sedentary behavior increased significantly over the course of pregnancy (*p* < 0.001). Post-hoc tests indicated differences in mean sedentary behavior at 16–18 weeks versus at 24–26 and 32–34 weeks but no differences from 24–26 weeks and 32–34 weeks (Figure 1). Physical activity levels significantly decreased over the course of pregnancy. Post-hoc tests indicated no differences in mean physical activity at 16–18 weeks versus at 24–26 weeks but differences from 16–18 versus 32–34 weeks and 24–26 versus 32–34 weeks (Figure 1).

**Figure 2:**
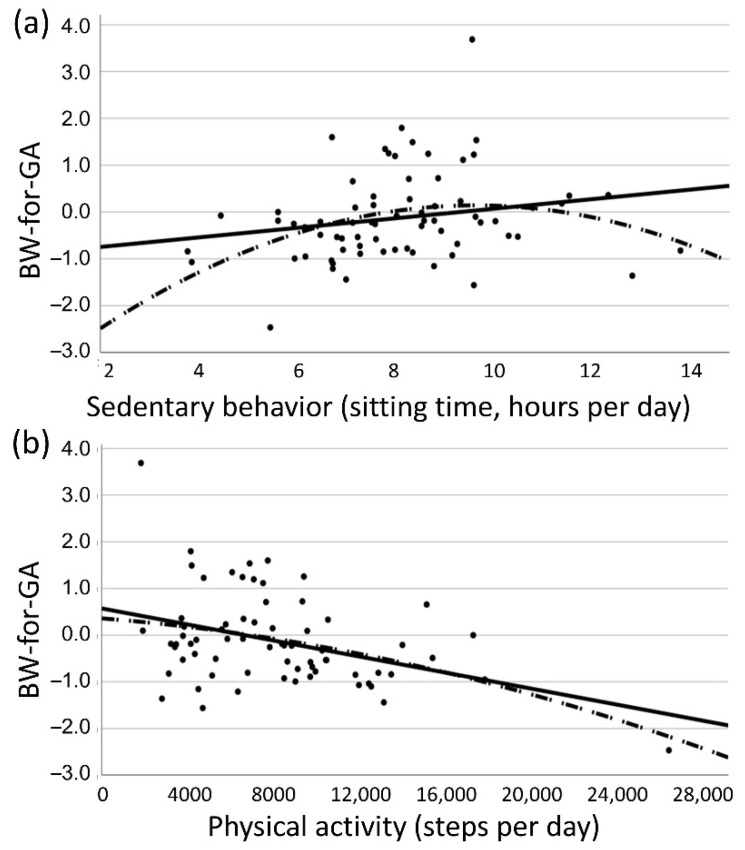
Relationships between sedentary behavior (**a**) and physical activity (**b**) at 16–18 weeks gestation with birthweight-for-gestational age Z-scores (BW-for-GA).

Results of linear regression analyses are shown in Table 2. Unadjusted models showed no linear relationships between sedentary behavior and BW-for-GA at any evaluation period. We observed a curvilinear relationship between sedentary behavior and steps per day at 16–18 weeks gestation and BW-for-GA. Birthweight-for-gestational age Z-scores were within normal-for-gestational age ranges but slightly lower among women with low and high levels of sedentary behavior (Figure 2). We observed a significant negative relationship between physical activity and BW-for-GA at 16–18 weeks and at 32–34 weeks gestation. Figure 2 shows the relationship at 16–18 weeks.

Table 3 shows results of multivariate models validating relationships between sedentary behavior and physical activity at 16–18 weeks gestation and BW-for-GA. The relationship between physical activity and BW-for-GA at 32–34 weeks did not persist when controlling for covariates (data not shown). The curvilinear relationship between sedentary behavior at 16–18 weeks (Model 1) and BW-for-GA persisted when controlling for covariates, as did the negative relationship between physical activity at 16–18 weeks (Model 2) and BW-for-GA. The full model, including covariates, physical activity, and sedentary behavior (Model 3), suggested that the curvilinear relationship between sedentary behavior and BW-for-GA was independent of physical activity levels.

As noted above, we re-ran analyses removing one outlier with very high birthweight (4800 g) and one outlier with high mean steps per day at 16–18 weeks (26,672). Results were unchanged (data not shown).

## 4. Discussion

Results of the current study demonstrate a modest curvilinear relationship between sedentary behavior at 16–18 weeks gestation and birthweight-for-gestational age Z-scores (BW-for-GA), suggesting slightly lower birthweights within normal ranges among infants of participants with both low and high levels of sedentary behavior. These relationships were independent of physical activity patterns and also key covariates, such as maternal age [15] and education [16], that are commonly associated with birthweight. Both physical activity and sedentary behavior during pregnancy might affect not only maternal health outcomes but also fetal development through effects on maternal physiological factors, such as changes to blood flow distribution and uteroplacental blood flow, glucose metabolism and availability for the fetus, and changes to stress hormones in response to activity patterns [17,18,19]. Some of these changes, such as higher fasting glucose and risk of insulin resistance observed in some studies of sedentary behavior [20], might be expected to result in increased risk of macrosomia. However, increased risk of lower birthweight with high levels of sedentary behavior is also plausible. For example, past studies show that greater sedentary behavior might be related to worse placental perfusion, which could negatively affect fetal growth and development [21]. On the other end of the spectrum, lower birthweights among infants of women with very low levels of sedentary behavior mirror past studies showing that prolonged standing might result in reduced intrauterine growth [22]. More detailed studies of sedentary behavior during pregnancy, its relationships with maternal and infant health outcomes, and the mechanisms underlying these relationships are needed.

Relationships between sedentary behavior and birthweight were evident early but not later in pregnancy. This might reflect greater sensitivity of the developing fetus to the physiological effects of sedentary behavior earlier during development or reduced variability in activity patterns over the course of pregnancy that, coupled with the small sample size here, limits statistical power to detect modest relationships. These differences over the course of pregnancy might underlie some inconsistencies in past studies, as many recruit women later in pregnancy, when relationships might be more difficult to detect.

A systematic review in 2017 highlighted 26 studies that evaluated sedentary behavior during pregnancy, 13 using device-assessed measures and 13 using questionnaire measures. Of these, only five assessed links with infant outcomes including birthweight [23]. We identified another six papers assessing relationships between sedentary behavior and birthweight. Results are mixed, with eight studies showing no associations, two showing negative relationships, and one showing increased risk for macrosomia with increased sedentary behavior. Below, we review characteristics and results of these studies.

### 4.1. Studies Showing no Relationships between Sedentary Behavior and Birthweight

We identified eight studies showing no relationships between sedentary behavior during pregnancy and birthweight. Studies in Spain of 94 participants showed no associations between accelerometer-assessed sedentary behavior in the early second trimester and birthweight. Mean time spent in sedentary behavior averaged 3598 min per week, or around 8.6 h per day [21]. Similarly, data from 111 participants from two cohort studies in the Netherlands [24] showed no associations between birthweight and accelerometer-assessed sedentary behavior at 15 weeks gestation or with change in sedentary behavior from 15 to 32–35 weeks. Sedentary behavior averaged 530 min (8.8 h) at 15 weeks and 505 min (8.4 h) at 32–35 weeks. Finally, studies in the UK of 140 participants with obesity in a dietary and physical activity intervention [25] showed no associations between accelerometer-assessed sedentary behavior at 16–18, 27–28, and 35–36 weeks gestation and macrosomia. Mean time spent in sedentary behavior ranged from 563–622 min (9.4–10.4 h) per day.

Other studies used self-reported estimates of sedentary behavior. Results of the Omega cohort study in the USA [26] showed no associations between self-reported leisure time sedentary behavior (non-work time spent sitting) in the year before pregnancy (*n* = 1373) and early pregnancy (*n* = 1535, mean 15 weeks) with mean birthweight. Dividing women into quartiles based on sedentary behavior, birthweight was lower as pre-pregnancy sedentary behavior increased, but these differences were not statistically significant (*p* = 0.11). On average, women reported 2.3 and 2.6 h per day in leisure sedentary behavior during pre- and early pregnancy, respectively. Similarly, studies in Denmark of 4458 healthy women delivering at term [27] suggested that self-reported participation in mostly sedentary leisure activities at around 16 or 30 weeks gestation did not predict mean birthweight or risk of low (<2500 g) or high (≥4500 g) birthweight compared to women with light or moderate to heavy leisure physical activity.

Data from prospective, population-based studies in Sweden [28] assessed self-reported sedentary behavior at 32–34 weeks among 2203 participants with singleton pregnancies via a validated question about hours per day sitting, not including sleeping. Sedentary time was not associated with mean birthweight, low birthweight, or macrosomia. Most participants (34.6%) reported from 4 to 6 h of sedentary time per day. Similarly, prospective cohort studies in India [29] included validated questionnaires to assess occupational activities, discretionary exercise, household chores, sedentary activities, hobbies, and sleep over 24 h in each trimester among 546 pregnant women. In the first trimester, participants in the highest physical activity tertile, with moderate/heavy physical activity, had increased odds of low birthweight compared to those in the first tertile, who were largely sedentary. However, these patterns were not evident in the second and third trimesters, and sedentary behavior itself was not an independent predictor of birthweight in any trimester. Sedentary behavior at baseline averaged 175 min (2.9 h).

Finally, case-control studies in Brazil [30] of 273 cases with low birthweight compared to 546 controls with normal birthweight used interviews following delivery to assess physical activity during a typical week in the second trimester, including housework, work outside home, leisure time, and transportation. Time spent in sedentary activities, classified as <2.4 h, 2.4 to <5 h, and ≥5 h, and time spent watching television (<1.5 h, 1.5 to <4 h, ≥4 h), did not predict low birthweight. Similarly, comparisons of women classified as sedentary (*n* = 539, or 65.8% of the sample), those classified as “little activity or active” (*n* = 123), and those classified as “very active” (*n* = 157) showed no relationships with birthweight.

### 4.2. Studies Showing Negative Relationships between Sedentarity and Birthweight

We identified two studies showing negative relationships between sedentarity and birthweight, both of which assessed self-reported activity patterns. The Avon Longitudinal Study of Parents and Children in the UK [31] included questionnaires in the first and second trimester assessing daily leisure, household, and occupational physical activities. Participants who reported “mostly sitting” were classified as having a sedentary lifestyle. Analyses of 11,737 singleton live births showed that a sedentary lifestyle in the first and second trimesters was modestly negatively associated with birthweight. Similarly, a prospective randomized controlled trial in Germany [32] of an intervention to improve prenatal weight development included evaluations of sedentary behavior at ≤12 weeks (*n* = 1904) and >29 weeks (*n* = 1890) gestation via the Pregnancy Physical Activity Questionnaire. Sedentary behavior at >29 weeks predicted lower birthweight, and sedentary behavior at both time points predicted increased odds of low birthweight but not macrosomia, small for gestational age, or large for gestational age. The same study showed a non-significant trend between sedentary behavior at ≤12 weeks (*p* = 0.051) and at >29 weeks (*p* = 0.070) and increased odds of preterm delivery.

### 4.3. Studies Showing Relationships between Sedentarity and Macrosomia

A prospective cohort study in Ireland [33] among 50 healthy pregnant women predicted to deliver infants with macrosomia and 50 healthy controls showed that women predicted to deliver a macrosomic infant spent more time in accelerometer-assessed sedentary behavior at 26–37 weeks gestation than controls (16.1 versus 13.8 standardized hours, including sleep time). Results were similar when comparing groups based on actual (rather than predicted) birthweights.

### 4.4. Comparisons of Results

Overall, studies of relationships between sedentary behavior and birthweight are inconclusive. Differences in sample characteristics, measurement methods, and timing of evaluations across pregnancy might underlie some of these differences. Of studies assessing objectively measured sedentary behavior, three suggest no significant relationships with birthweight [21,24,25]. One suggests increased risk of macrosomia, but other outcomes, such as low birthweight or continuous relationships between sedentarity and birthweight, were not assessed [33]. As in the current study, these are limited by relatively small sample sizes (ranging from 94–140). Studies using self-report measures are no more conclusive, with two showing negative relationships between birthweight and sedentarity [31,32] and five showing no relationships [26,27,28,29,30]. The lower birthweights among women with high levels of sedentarity in the current analyses are similar to those in studies reporting negative relationships between self-reported sedentary behavior and birthweight [31,32] and to non-significant trends observed in other studies [26]. It is not clear if curvilinear relationships were assessed in these studies, and we might postulate that modest curvilinear relationships could be masked due to imprecisions in self-report measures of sedentary behavior. Overall, results suggest that if there are indeed relationships between sedentary behavior during pregnancy and birthweight, they are modest. Results of the current study suggest that relationships are strongest in early pregnancy, and analyses later in pregnancy might not be able to detect such modest effects. Furthermore, past studies tend to assess linear relationships, whereas our results highlight the importance of evaluating curvilinear relationships.

### 4.5. Relationships between Physical Activity and Birthweight

Relationships between physical activity and birthweight have been well studied. Our measure of physical activity, steps per day, provides only a basic indicator intended to allow us to refine our analyses of sedentary behavior. This measure does not reflect activity intensity or capture some activities, such as swimming, that might be recommended or practiced during pregnancy. Results might be more or less pronounced for other measures, such as light, moderate, and vigorous activity levels. As such, results of the current study do not contribute new insights into this topic, but are consistent with many other studies. Meta-analyses are conclusive that regular physical activity during pregnancy does not adversely affect birthweight in healthy, low-risk pregnant women, and given the benefits of physical activity for other maternal physical and mental health outcomes, remaining active during pregnancy should be encouraged [5,19,34,35].

The modest negative relationship observed here has been observed in many other studies. Meta-analyses [19] of 37 observational studies identified eight (21.6%) that showed negative relationships between physical activity during pregnancy and infant birthweight, 25 (67.6%) that showed no relationships, and four (10.8%) that showed positive relationships. Furthermore, 15 studies comparing participants with “low” versus “high” physical activity levels suggested a U-shaped relationship between physical activity and birthweight such that high physical activity levels predicted lower birthweight, whereas “moderate” physical activity levels predicted higher birthweight. Other meta-analyses [5] of 28 randomized controlled trials of structured exercise interventions showed that maternal physical activity predicted a modest yet significant reduction in birthweight and reduced risk of macrosomia or large for gestational age, with no increased risk for low birthweight or small for gestational age. Similarly, meta-analyses [34] of 14 randomized controlled trials of exercise interventions among healthy sedentary or inactive women with low-risk pregnancies indicated a modest yet significant reduction in birthweight, suggesting a shift of birthweight within the normal range. Finally, meta-analyses [35] of 73 observational and experimental studies showed that prenatal exercise was not associated with birthweight, low birthweight, small for gestational age, or intrauterine growth restriction but predicted reduced risk of macrosomia.

It is possible that there is a threshold at which high levels of physical activity hold relevant risks for low birthweight or small for gestational age, and authors of systematic reviews highlight that the identification of this threshold through more detailed studies remains necessary [19], especially given that small for gestational age has been less frequently assessed than macrosomia in many trials [5]. Based on the independent relationships between physical activity and sedentary behavior with birthweight observed here, consideration of these behaviors simultaneously might represent a research priority in the identification of thresholds. Interactions between sedentary behavior and physical activity might predict particularly increased risk among some people, such as those with very low or high physical activity levels coupled with very low or high levels of sedentary behavior. Such studies will likely require objective measurement of activity patterns in relatively large samples.

### 4.6. Strengths, Limitations, and Future Directions

This study is limited by the sample size, which limits generalizability and statistical power. The use of flyers in the waiting room might have resulted in a sample biased toward participants more interested in or familiar with research studies. It is also possible that participants modified their behavior in response to the observation period. We might expect that this would result in lower levels of sedentary behavior and higher levels of physical activity compared to their normal patterns, although activity patterns observed here are similar to those in other studies. Furthermore, physical activity and sedentary behavior were evaluated over the course of three days at each assessment period. While past studies show that three valid days of measurement agree with measurements over the course of four days or more [36], a longer evaluation period might provide a more nuanced perspective. Finally, data collection was typically on weekdays, and we cannot account for potential differences in activity patterns on weekdays versus weekends. These factors would not be expected to systematically bias relationships between activity patterns and birthweight but might limit generalizability.

Detailed medical records were not available for all participants, and we were thus unable to control for factors such as pre-pregnancy activity patterns, body mass index, gestational diabetes, or gestational weight gain that are associated with physical activity and sedentary behavior during pregnancy. Furthermore, data on other behaviors, such as sleep and diet, that might be correlated with physical activity and sedentary behavior were not assessed here. Future studies with larger sample sizes that permit analyses of interactions between these factors would be an interesting addition to the literature.

This study is strengthened by the prospective longitudinal data collection, which allowed us to evaluate sedentary behavior over the course of pregnancy. The objective measures of activity patterns and collection of data on birthweight from medical records is another strength. Finally, the sample is diverse in terms of sociodemographic factors, such as education, income, ethnic background, and immigration status. Recruiting diverse samples is a priority given sociodemographic variations in sedentary behaviors in the general population and during pregnancy.

Future analyses in early pregnancy with larger samples and consideration of factors, such as maternal weight, other health behaviors, and illnesses or conditions that might affect or interact with activity patterns, are a priority. As noted above, a U-shaped relationship between physical activity and birthweight has been documented in many studies, and based on results of the current study, similar non-linear relationships should be investigated for sedentary behavior. Past case-control studies of 1166 participants in the USA have shown a U-shaped relationship between self-reported television viewing during pregnancy and preterm birth such that odds for preterm birth were highest among those with both low (<15 h/week) and high (>42 h/week) viewing time [37]. This observation is consistent with the pattern observed here for birthweight. Finally, more detailed studies among at-risk and underrepresented women, with objective measures of activity patterns across the course of pregnancy, remain necessary [5,19].

## 5. Conclusions

Overall, results of the current study and past studies suggest that relationships between sedentary behavior and birthweight are likely to be modest. However, given the high levels of sedentary behavior observed during pregnancy in many studies and the associated risks to maternal mental and physical health [23], even modest associations with birthweight merit further consideration. More detailed studies assessing objectively measured activity patterns among diverse samples could help to guide the development of concrete recommendations on sedentary behavior during pregnancy and, ultimately, the development of more comprehensive interventions to improve maternal and infant health.

## Figures and Tables

**Table 1:** Sample characteristics.

	Mean (SD), Median (IQR), or *n* (%)	Range
Maternal characteristics		
Age (y), mean (SD)	31.3 (6.0)	19 to 45
Number of children, median (IQR)	1.0 (2)	0 to 5
Education, *n* (%)		
Secondary	23 (32.4)	
College	17 (23.9)	
University	31 (43.7)	
Household income, *n* (%)		
<$20,000	23 (32.4)	
$20,000–50,000	34 (47.9)	
>$50,000	14 (19.7)	
Immigrant, *n* (%)	47 (66.2)	
Visible minority, *n* (%)	48 (67.6)	
Sedentary behavior (sitting time, h/day), mean (SD)		
16–18 weeks	8.0 (1.9)	3.8 to 13.7
24–26 weeks	8.8 (1.7)	5.7 to 13.4
32–34 weeks	8.9 (2.0)	4.3 to 14.5
Physical activity (steps per day), mean (SD)		
16–18 weeks	8172 (4356)	1842 to 26,672
24–26 weeks	7805 (3546)	1949 to 19,842
32–34 weeks	6416 (3059)	593 to 15,762
Infant characteristics at birth		
Sex, *n* (%)		
Boy	41 (57.7)	
Girl	30 (42.3)	
Birthweight (grams), mean (SD)	3361 (476)	1350 to 4800
Gestational age (GA) at birth (weeks), mean (SD)	39.4 (1.4)	34 to 42
Birthweight-for-GA Z-score (BW-for-GA), mean (SD)	−0.12 (0.96)	−2.47 to 3.69

**Table 2:** Results of simple linear regression models testing relationships between (**a**) sedentary behavior and (**b**) physical activity with birthweight-for-gestational age Z-scores.

a. Sedentary Behavior	*p*-Value	R^2^
Evaluation #1 (16–18 weeks)		
Sedentarity	0.093	0.040
Sedentarity Squared (curvilinear)	0.021	0.073
Evaluation #2 (24–26 weeks)		
Sedentarity	0.729	0.002
Sedentarity Squared (curvilinear)	0.104	0.038
Evaluation #3 (32–34 weeks)		
Sedentarity	0.458	0.008
Sedentarity Squared (curvilinear)	0.317	0.015
**b. Physical Activity (PA)**	***p*-Value**	**R^2^**
Evaluation #1 (16–18 weeks)		
Physical activity	0.001	0.148
PA Squared (curvilinear)	0.477	0.006
Evaluation #2 (24–26 weeks)		
Physical activity	0.850	0.001
PA Squared (curvilinear)	0.322	0.014
Evaluation #3 (32–34 weeks)		
Physical activity	0.045	0.057
PA Squared (curvilinear)	0.689	0.002

**Table 3:** Results of multiple regression models testing relationships between sedentary behavior and physical activity at 16–18 weeks gestation with birthweight-for-gestational age Z-scores. β indicates standardized coefficients.

	Model 1			Model 2			Model 3		
	β	*p*-Value	R^2^	β	*p*-Value	R^2^	β	*p*-Value	R^2^
Age (y)	0.02	0.889	0.071 *	0.01	0.962	0.071 *	−0.01	0.973	0.071 *
No. children	0.09	0.563		0.17	0.251		0.17	0.254	
Education	−0.10	0.423		−0.11	0.390		−0.11	0.369	
Household income	−0.15	0.288		−0.14	0.299		−0.17	0.203	
Immigration status	0.16	0.320		0.12	0.434		0.11	0.471	
Visible minority status	0.22	0.166		0.21	0.161		0.22	0.127	
Sedentarity	1.73	0.012	0.027	---	---	---	1.21	0.067	0.027
Sedentarity Sq. (curvilinear)	−1.58	0.020	0.076	---	---	---	−1.32	0.040	0.076
Physical activity	---	---	---	−0.40	0.001	0.151	−0.43	0.004	0.109

* R^2^ for all covariates together.

## Data Availability

Data are available from the corresponding author, K.N.D., upon request.
